# Nuts and Non-Alcoholic Fatty Liver Disease: Are Nuts Safe for Patients with Fatty Liver Disease?

**DOI:** 10.3390/nu12113363

**Published:** 2020-11-01

**Authors:** Maria Corina Plaz Torres, Giorgia Bodini, Manuele Furnari, Elisa Marabotto, Patrizia Zentilin, Edoardo G. Giannini

**Affiliations:** Gastroenterology Unit, Department of Internal Medicine, University of Genoa, IRCCS-Ospedale Policlinico San Martino, 16132 Genoa, Italy; maco.plaz87@gmail.com (M.C.P.T.); giorgia.bodini@unige.it (G.B.); manuele.furnari@unige.it (M.F.); elisa.marabotto@unige.it (E.M.); pzentilin@unige.it (P.Z.)

**Keywords:** steatosis, steatohepatitis, liver disease, hepatocellular carcinoma, aflatoxin, peanuts

## Abstract

Diet and lifestyle interventions are the recommended treatment for patients with non-alcoholic fatty liver disease (NAFLD), with the aim of achieving a 7–10% weight loss. Several dietary patterns have been suggested for this purpose, however, to date, the best one is represented by the Mediterranean diet (MD) as it is rich in macro- and micro- nutrients known for their effectiveness in health-promotion and cardio-vascular disease prevention. Moreover, MD is characterized by the inclusion of nuts. These foods have shown potential benefits in health-promotion as they are rich in fibers, which have lipid-lowering effects, rich in mono- and poly-unsaturated fatty acids, which help reduce insulin-resistance and serum cholesterol, and contain anti-oxidants which reduce oxidative stress and inflammation. Additionally, nuts are associated with a better control, or reduction, of Body Mass Index (BMI). All these effects are useful targets to achieve in NAFLD, so that nuts have been proposed as a suitable dietary treatment supplement for weight and metabolic control in these patients. In recent years, health authorities raised an alert on nuts consumption as these may be at high risk of aflatoxin (AF) contamination, for which controls and legislations are different among countries. AF is a well-known cancerogenic agent and a recognized risk factor for hepatocellular carcinoma. Patients with NAFLD have an overall, inherent sevenfold increased risk of developing hepatocellular carcinoma as compared with the general population. In this context, one could argue that recommending the inclusion of nuts in the diet of NAFLD patients has to be balanced with the risk of potential chronic exposure to AF, and every effort should be pursued to assure the safety of these nutrients. In this review, we aim to summarize the benefits of nuts consumption, the evidence for AF contamination of nuts and the consequent potential risks in patients with NAFLD.

## 1. Introduction

Non-alcoholic fatty liver disease (NAFLD) is the hepatic manifestation of the metabolic syndrome and is defined as the accumulation of fat in the liver in patients who do not consume excessive amounts of alcohol (i.e., more than 20 g/day for women and less than 30 g/day for men). NAFLD encompasses different clinical scenarios, from the simple accumulation of fat in the liver (steatosis), to non-alcoholic steato-hepatitis (NASH), cirrhosis and its complications [1]. Even though the development of cirrhosis and its complications is relatively uncommon [1], NAFLD is a recognized public health issue since its prevalence worldwide is extremely high. Indeed, the disease affects approximately 25% of the global population, with regional differences, and peaks as high as 46% in the United States of America [2]. Accordingly, NASH-related end-stage liver disease and hepatocellular carcinoma (HCC) are the most rapidly growing indications for liver transplantation and actually represent the second leading indication for liver transplantation in the USA [3]. Nowadays, several drugs are under investigation for the treatment of NAFLD but most trials are still open and no drug has officially been approved and validated. Therefore, the only recommended treatment consists of lifestyle and dietary interventions targeting weight loss of around 7–10% of the baseline weight [4]. Different dietetic regimens have been proposed for this purpose, but none has adequately been evaluated in randomized controlled trials including large cohorts of patients. This notwithstanding, the Mediterranean diet (MD) seems to be the best dietary pattern for the NAFLD population as it is poor in sugars and saturated fatty acids and rich in macro- and micro-nutrients known for their effectiveness in health-promotion and cardio-vascular disease prevention [5,6,7]. Notably, the MD has also shown efficacy in reducing hepatic fat content and NASH severity [7,8,9,10]. In particular, the MD contains a low proportion of meat and dairy products while being rich in fibers, fish, seafood, olive oil and nuts, which are the main source of added fat [11]. While the beneficial effects of the MD components have been extensively reported in the general population [5,6,12], there is still limited information regarding the potential benefit of its components, including nuts, in patients with NAFLD. In this review, we aim to summarize the beneficial effects and potential negative implications of nuts consumption, as an integral part of the MD in patients with NAFLD.

## 2. Nut Consumption: Beneficial Metabolic Effects

The term “nuts” is used to generally define a group of several dry, edible, energy-dense fruits or seeds including walnuts, pistachios, almonds, hazelnuts, chestnuts and peanuts that share a similar nutritional profile. Indeed, these foods are rich in fibers, mono- and poly-unsaturated fatty acids (MUFAs and PUFAs) such as omega-3 fatty acids, minerals, essential amino-acids, vitamin E, B2 and B9, antioxidants and phenolic compounds [13]. These nutrients are health-promoting as they induce positive metabolic effects (Table 1) which help prevent cardio-vascular diseases and cardio-vascular events [14,15], mainly coronary heart disease. Importantly, cardio-vascular events are the major cause for mortality among the NAFLD population [16], especially for patients with NASH or more advanced stages of fibrosis [17]. 

In particular, due to the high content in fibers, MUFAs, PUFAs and phytosterols, nut consumption can favorably alter lipid profiles by significantly reducing total cholesterol, low-density lipo-protein cholesterol (LDL-C) and triglycerides (TG), without altering the serum concentration of high-density lipo-protein cholesterol (HDL), and independently from the background diet and from the type of nuts [18,19]. Indeed, a meta-analysis including 61 trials with intervention duration ranging from 3 to 26 weeks, showed that nut intake (per serving/day) lowered total cholesterol (−4.7 mg/dL; 95% CI: −5.3, −4.0 mg/dL), LDL cholesterol (−4.8 mg/dL; 95% CI: −5.5, −4.2 mg/dL), ApoB (−3.7 mg/dL; 95% CI: −5.2, −2.3 mg/dL), and triglycerides (−2.2 mg/dL; 95% CI: −3.8, −0.5 mg/dL) with no statistically significant effects on other outcomes (HDL-cholesterol) [18]. These effects were dose-related (i.e., higher as the daily servings of nuts increased, stronger for servings ≥60 g/day) [18] and mediated via a reduction of cholesterol absorption, the inhibition of the enzyme β-hydroxy β-methylglutaryl-CoA (HMG-CoA) reductase and an enhancement in the production of bile acids [14]. A recent randomized controlled trial reported similar results and showed that a high daily almond consumption acts synergistically in further reducing non HDL-cholesterol levels in patients already under statin treatment [20]. Moreover, a recent meta-analysis showed that peanuts intake has a positive significant effect on HDL cholesterol reduction while it has no effect on LDL, triglycerides and weight [21].

The inclusion of nuts in the diet may also lead to a better glycemic control [22] but findings in this field are not consistent. Indeed, in a meta-analysis including 12 randomized controlled trials, diets which implemented tree nuts intake at a median dose of 56 g/day significantly lowered HbA1c (−0.07% (95% CI:−0.10, −0.03%); *p* = 0.0003) and fasting glucose (−0.15 mmol/L (95% CI: −0.27, −0.02 mmol/L); *p* = 0.03) as compared with control diets. However, no significant treatment effects were observed for fasting insulin and homeostasis model analysis for insulin resistance (HOMA-IR), although the direction of trends favored tree nuts consumption [23]. Of note, the main limitation of this meta-analysis is the short duration and poor quality of the majority of trials included. Nevertheless, further studies found nuts consumption, in particular peanuts [24,25], pistachios [26,27] and almonds [28,29,30], to reduce post-prandial glycemic responses, basal glycemia and HOMA-IR in patients with type-2 diabetes mellitus or pre-diabetes. Similarly to the effects on lipid control, the improvement in glycemic control seems to be dose-dependent and mainly mediated via the substitution of carbohydrates with MUFAs and PUFAs that induce glucagon-like peptide-1 (GLP-1) release from pancreatic beta-cells and insulin-like growth factor 1 (IGF-1) gene expression and release from hepatocytes, via a peroxisome proliferator-activated receptor α (PPAR α)-dependent pathway [31,32,33,34,35]. 

Regular nut intake may be beneficial for weight loss and appetite control as well. Indeed, in a study including 65 overweight and obese subjects with metabolic syndrome features, who were randomized to consume an almonds-enriched low-calorie diet or a self-selected complex carbohydrates low-calorie diet, the group assigned to the almonds-enriched low-calorie diet experienced a sustained and greater weight reduction for the duration of the 24-week intervention [36]. Indeed, patients assigned to the almonds-enriched arm achieved a body mass index (BMI) decrease of 18% versus the 11% BMI decrease in the self-selected low-calorie diet group (*p* < 0.0001). Similarly, in the PREDIMED study, the inclusion of 30 g/day of nuts or 50 mL/day of extra-virgin olive oil to a background MD for 3 years led to a significant reduction in body weight that was highest in the two lowest quintiles and in the highest quintile of change in nut/olive oil consumption from the baseline diet [37]. Particularly, body weight decreased nearly 5 kg in the two lowest quintiles and in the highest quintile of nuts/olive oil consumption. In the two middle quintiles, the body weight decreased approximately 2 kg, but the change was still significant. The increased energy density was therefore not associated with body weight gain [37]. Conversely, a study on 30 heathy, non-obese participants, randomized to either supplement the diet with fruits or nuts, each at +7 kcal/kg bodyweight/day for two months, showed a significant body-mass index (BMI) increase in both groups [38]. However, notwithstanding the BMI increase, the basal metabolic rate only increased in the nuts-group [38]. Overall, most evidence from investigational studies on the effects of nut consumption on body weight found an inverse association [36,37] or no association [39,40,41] between nut intake and body weight [14]. Moreover, regular intake of nuts as a snack reduces appetite [42,43] via an induction of cholecystokinin (CCK) and GLP-1 and peptide YY (PYY) secretion [44], which is related to their high content in MUFAs and PUFAs. 

In summary, regular nut intake induces an amelioration in lipid metabolism, may help glycemic control and helps weight reduction; all effects which are main targets in the dietetic treatment of NAFLD.

## 3. Nut Consumption and Cardio-Vascular Disease

Taking into account all of the abovementioned metabolic effects of nuts, the increasing promotion of nuts as a nutritional support for the prevention of cardio-vascular events is not surprising. Accordingly, nuts are frequently recommended to be included into the dietary treatment of NAFLD [45], not only with the aim of ameliorating lipid and glycemic metabolism but also with the aim of reducing the risk of cardio-vascular morbidity and mortality which represent the major cause for mortality among the NAFLD population [3].

The cardio-protective effects of nuts are supposed to be a consequence of the synergistic effect of the amelioration in lipid metabolism and weight control and the antioxidant properties of the phenolic compounds which can modulate nitric oxide production, thereby acting on vascular function [14,15]. Moreover, nut consumption affects the composition and function of the gut microbiota leading to a reduced proportion of bacterial species whose metabolites have atherogenic properties [46,47,48]. Additionally, nut intake increases the proportion of probiotic and butyrate-producing species which are associated with reduced microbially derived, proinflammatory secondary bile acids and LDL cholesterol [46,47]. Finally, regular nut consumption may also lead to a reduction in circulating levels of pro-inflammatory markers such as erythrocyte sedimentation rate (ESR), interleukin-6 (IL6), tumor necrosis factor-alpha (TNF-α) and C-reactive protein [49,50,51,52] which are associated with higher risk of cardio-vascular mortality and are usually elevated in NAFLD and implicated in its pathogenesis [53].

In the last 10 years, a growing body of evidence has confirmed the cardio-protective effect of nuts. Indeed, different cohort studies including large numbers of participants from different geographical regions obtained similar results on nuts-related reduction of the incidence of—and mortality from—cardio-vascular diseases in subjects with and without pre-existing cardio-vascular risk factors [54,55,56,57,58,59,60,61]. In the PREDIMED multi-center trial, which compared the rates of major cardio-vascular events among 7447 subjects with high cardio-vascular risk at baseline, randomized to either an MD supplemented with extra-virgin oil or nuts, or a control diet (reduced fat diet), the incidence of major cardio-vascular events was significantly lower among those assigned to the either olive oil- or nuts-supplemented MD [54]. Similarly, in a large cohort study by Guasch-Ferre et al. including more than 200,000 US men and women, total nut consumption was inversely associated with total cardio-vascular disease and coronary heart disease (after adjustment for cardio-vascular risk factors) [56]. In particular, consumption of peanuts and tree nuts (two or more times/week) and walnuts (one or more times/week), was associated with a 13–19% lower risk of total cardio-vascular disease and 15–23% lower risk of coronary heart disease [56]. The same group analyzed the association of within-individual changes in consumption of total and specific types of nuts and the subsequent risk of incident cardio-vascular disease (CVD) during a follow up interval of up to 26 years [57]. Compared with individuals who remained non-consumers in a 4-year interval, those who had a higher consumption of total nuts (≥0.5 servings/day) had a lower risk of CVD (relative risk (RR), 0.75; 95% CI, 0.67–0.84), coronary heart disease (RR, 0.80; 95% CI, 0.69–0.93), and stroke (RR, 0.68; 95% CI, 0.57–0.82) in the following 4 years [57]. Increasing intakes of tree nuts (RR, 0.90; 95% CI, 0.85–0.96), walnuts (RR, 0.83; 95% CI 0.73–0.93), and peanuts (RR, 0.91; 95% CI 0.85–0.97), per 0.5 servings/day, were each significantly associated with lower risk of CVD. Increase in consumption of tree nuts (RR 0.90, 95% CI 0.83–0.98) and peanuts (RR 0.90; 95% CI 0.83–0.98) per 0.5 servings/day, was also associated with a lower risk of coronary heart disease. An increase in walnut consumption was associated with lower risk of stroke (RR 0.80; 95% CI 0.67–0.95) [57]. These findings are in accordance with previous cohort studies [55,56,59,60,61,62] and meta-analytic evidence [63,64,65] showing a dose-dependent decreased risk of CVD and cardio-vascular mortality in nut consumers as compared with non-consumers, with hazard ratios for cardio-vascular mortality as low as 0.61 (95% CI: 0.42, 0.91) [64]. Notably, the protective effect of nuts in cardio-vascular prevention has been demonstrated also independently from the socio-economic status and ethnicity and independently from the type of nut, including peanuts [58,59].

These findings are relevant as nuts are a relatively expensive food in most Western countries. Hence, consumption of peanuts, given their general affordability, may be considered a cost-effective measure for preventing cardio-vascular diseases. Of note, the protective effects of nuts are not independent from their processing. Indeed, when considering peanuts, cardio-vascular-related diseases and mortality are only reduced by the consumption of the whole product (either raw or roasted) but not by peanut butter [58,59,62]. This finding is probably related to the fact that peanut butter consumers are more likely to consume red meat, to currently smoke cigarettes and exercise less, all factors that may outweigh the beneficial potential of peanuts [62]; besides, the processing of peanut butter may alter the nuts properties thereby reducing their beneficial effects [62,66]. 

A generally healthier lifestyle was associated with nut consumption in most studies, mainly carried out in Western countries. Therefore, one could argue that the better outcomes for nut-consumers may be the result of a more complex interaction between diet and daily habits, and not a direct effect of nut consumption. In order to investigate the correlation between nuts and mortality, independently from lifestyle, Eslamparast et al. conducted a large population-based cohort study including 50,045 subjects in Iran, where nut consumption is extremely common and does not correlate with a healthier lifestyle [67]. Overall, this study showed that nut consumption was inversely associated with all-cause mortality during a 7-year period of follow up [67]. The pooled multivariate adjusted hazard ratios for death among participants who ate nuts, as compared with those who did not, were 0.89 (95% confidence interval (CI), 0.82–0.95) for the consumption of less than one serving of nuts per week, 0.75 (95% CI, 0.67–0.85) for one to less than three servings per week and 0.71 (95% CI, 0.58–0.86) for three or more servings per week (*p* < 0.001 for trend). Among specific causes, significant inverse associations were observed between nut consumption (for both peanuts and tree nuts) and deaths due to cardio-vascular disease, all cancers and gastrointestinal cancers [67].

Overall, this evidence suggests that regular nut consumption is effective in the prevention for risk factors for cardio-vascular disease such as diabetes, dyslipidemia and obesity, and in the prevention of cardio-vascular diseases and cardio-vascular mortality, which is the major cause for death in patients with NAFLD. On these bases, it is of note that nuts are being implemented in the diets suggested for patients with NAFLD although specific studies aimed at determining the effects of nut consumption in liver-related events in this particular population are lacking [45,68]. 

## 4. Nuts and NAFLD

Reports from studies on the potential association between nuts and liver-related endpoints in patients with NAFLD are scanty and provided inconsistent results. Indeed, two recent cohort studies from Eastern countries investigated the association between nut consumption and NAFLD in the general population [69,70]. In both studies, regular nut intake was significantly and inversely associated with the risk of NAFLD, and the risk for NAFLD across categories of nut consumption showed a nearly 10% decrease as the weekly servings increased from <1 time/week to ≥4 times/week (for the latter, odds ratio (OR) 0.80, 95% confidence interval 0.69–0.92, *p* value < 0.01) [70]. A recent cohort study carried out in Korea reported that low intakes of vitamin C (odds ratio (OR), 4.23), vitamin K (OR, 3.93), folate (OR, 3.37), omega-3 fatty acids (OR, 2.16), and nuts and seeds (OR, 3.66) were associated with a significantly higher risk for developing NAFLD in the male cohort whereas in women, vitamin K (OR, 2.54) and vegetable (OR, 4.11) intakes showed a significant beneficial effect for lowering the NAFLD risk [71]. Similarly, Cueto-Galàn et al. analyzed the effects of nuts in a cohort of patients from the PREDIMED study and showed that the inclusion of nuts into an MD pattern improved the fatty liver index, a non-invasive marker of hepatic fat content, at 3–5 and 6-year follow up [72]. Different results were obtained by Bowen et al. who found no association between almond consumption and liver fat concentration (as measured by proton magnetic resonance spectroscopy) in a single-center, 8-week, randomized, controlled trial including 76 adults with elevated risk of type 2 diabetes who were assigned to daily consumption of either 2 servings of almonds (i.e., 56 g/day) or an isocaloric higher carbohydrate biscuit snack [73]. These results were consistent with a previous report showing no decrease in hepatic fat content (HFC) in a group of healthy subjects following a nut-enriched diet [38]. However, it has to be emphasized that the apparently negative studies included smaller cohorts of patients and had a shorter follow-up as compared with the study by Cueto-Galàn et al., that also had the merit of including nut consumption into an MD-based dietetic regimen [72].

All in all, the results of these studies surmise that nut consumption seems to decrease the risk for NAFLD in the general population, although its effects on liver-related outcomes among patients with a pre-existing diagnosis of NAFLD still has to be characterized by targeted prospective studies. 

## 5. Potential Harms of Nut Consumption in the NAFLD Population: Aflatoxin Contamination

**Figure 1:**
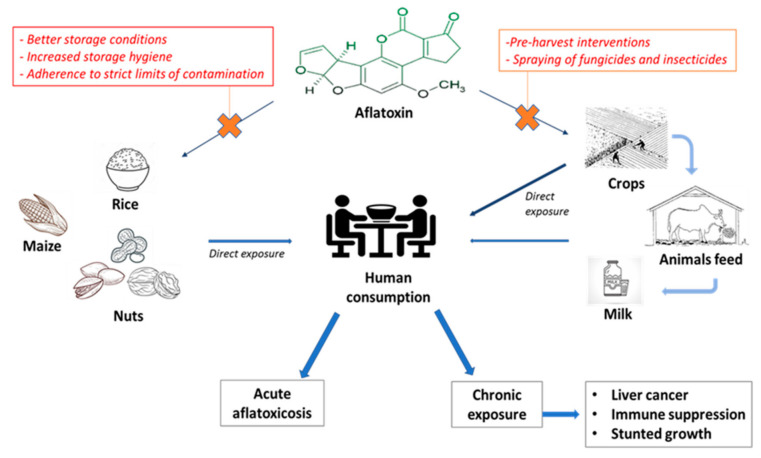
Pontential harms of aflatoxin exposure and possible strategies to prevent aflatoxin contamination of crops.

Despite the large evidence regarding the health-promoting effects of regular nut intake, there is a need to emphasize that these foods are also widely recognized to be contaminated by aflatoxins (AF), a secondary fungal metabolite largely found in other agricultural products as well, such as animal feed, milk, rice, cereals and maize [74,75,76] as summarized in Figure 1. According to the Food and Agriculture Organization (FAO, Rome, Italy), up to 25% of the foodstuffs are contaminated with mycotoxins on a global scale, although the exact proportion of the amount of foodstuffs that are treated or destroyed is actually not reported [77]. Among them, AF-B1 carries the highest genotoxic and carcinogenic effects; indeed, chronic AF-B1 exposure is a well-established risk factor for primary hepatocellular carcinoma and AF-B1 is listed among the group 1 carcinogenic agents in the 2002 report from the International Agency for Research on Cancer (IARC, Lyon, France) [78]. Consistent with this, the Codex Alimentarius Risk Analysis system, established by the FAO and the World Health Organization (WHO, Geneve, Switzerland), have set precise limitations for the allowed concentrations of aflatoxin contamination among food supplies, with the aim of minimizing human AF-B1 exposure [79]. However, the cut-offs for aflatoxin contamination differ among geographical areas. Indeed, in the European Union (EU) the European Commission (EC, Bruxelles, Belgium) regulation No. 1881/2006 imposed a maximum limit of 2 µg/kg for AF-B1 and 4 µg/kg for total AF contamination for peanuts and cereals that are intended for direct human consumption, the respective limits for almonds and pistachios are 8–10 µg/kg for AF-B1 and total AF, respectively, and 5–10 µg/kg, for AF-B1 and total AF respectively, for hazelnuts and Brazilian nuts [80]. This limit is set up at 20 µg/kg for the overall AF contamination of nuts (i.e., considering AF-B1, B2, G1 and G2) in the US [81] and at 5 µg/kg for AF-B1 in Iran [82]. In China, the maximum limits for AF contamination for peanuts and peanut products have been set at 20 µg/kg [83]. These limits have mostly been defined based upon the following risk estimation calculations: the *margin of exposure* (MOE) approach, proposed by the European Food Safety Authority (EFSA, Parma, Italy) [84], and/or the *quantitative liver cancer risk approach* proposed by the FAO and the WHO [85]. The MOE approach uses a reference point which corresponds to a dose that causes a low but measurable (1–10%) increase in tumor formation above background levels in experimental animals. This reference point is then compared with various estimated daily intakes (EDI) in humans, taking into account differences in consumption patterns [84]. The EFSA Scientific Panel on Contaminants in the Food Chain proposed to use the benchmark dose lower confidence limit for 10% extra risk (BMDL10) for characterizing the MOE, which represents the ratio between the BMDL10 and the EDI [84]. If the BMDL10 is used as the parameter to calculate the MOE, the EDI would be considered of concern from the public health point of view if the MOE value is lower than 10,000. MOE values do not quantify the risk of cancer incidence but only indicate a level of concern, [84] whereas the *quantitative liver cancer risk approach* formula considers the estimated number of liver cancer cases in one year and is based on multiplying the carcinogenic potency (expressed in a number of cancers per year per 100,000 individuals per ng of AF-B1 per kg of body weight per day) by the total intake of AF-B1 (expressed in ng of AF-B1 per kg of body weight per day) [85]. This formula distinguishes the carcinogenic potency for people who suffer from hepatitis B and for people without hepatitis B as it is known that hepatitis B virus could synergistically increase the chance of getting AF-B1-induced liver cancer [86]. 

These estimates, on the basis of which the maximum consented AF contamination limits have been established internationally, however, were calculated based upon data derived from the general population and take into account hepatitis B virus (HBV) prevalence. Hence, such limits are recommendable for the general population with/without HBV infection but whether such limits are to be considered safe also among subjects with an underlying fatty liver disease is unknown. Furthermore, if nut-supplemented diets are to be recommended for subjects with fatty liver disease, one could argue that these subjects may be at higher risk of AF-B1-induced HCC incidence as NAFLD patients do carry an inherent higher risk of developing HCC, as compared to the healthy general population [87]. For instance, NAFLD patients have an independently 7-fold increased risk of HCC in comparison to the general population, which further increases as fibrosis stages progress being higher in NASH patients than in NAFLD patients and highest in subjects with NASH-related cirrhosis [88,89]. Therefore, it seems reasonable to question whether the chronic exposure to AF-B1 related to a nut-supplemented diet, even within the limits recommended by the Food Authorities, is safe in this particular group of patients. Several genetic, metabolic and environmental modifiers, such as diet or lifestyle, contribute to the development of NASH-related HCC [90]. NASH–HCC pathogenesis is complex and incompletely understood and includes mechanisms involved in immune and inflammatory responses, DNA damage, oxidative stress and autophagy [90]. AF-B1-induced carcinogenesis seems to be secondary to mutations in the tumor-suppressor gene p-53 and to the activation of proto-oncogenes [91]. Moreover, chronic AF exposure can lead to immune suppression as well [92], which could potentially further increase the risk of cancer development [93]. Additionally, in vitro and animal studies have shown that AF-B1 hepatotoxicity is enhanced by exposure to the bacterial-derived endotoxin lipo-poly-saccharide (LPS), through mechanisms that are not completely clear but that involve the activation of inflammatory cells and the production of soluble inflammatory mediators, such as TNF-α [94,95]. Moreover, there is evidence that chronic exposure to some mycotoxins, including AF-B1, may alter the intestinal barrier integrity via the disruption of the tight junctions [96]. Tight junctions are one of the protein complexes that regulate intestinal paracellular permeability. In NAFLD and NASH, a significantly increased gut permeability has been demonstrated [53]; therefore, these patients are exposed at a higher risk of lipo-poly-saccharidemia which in turn promotes insulin resistance, obesity, hepatic fat accumulation and NASH development and progression [53]. In this context, a greater cause for concern can be posed relating to the safety of AF exposure in the NAFLD population. Indeed, if patients with NAFLD are exposed to higher levels of LPS as compared to the general population, then they are also at higher risk of AF-induced LPS-enhanced hepatotoxicity and liver disease progression. 

In some countries, such as Indonesia, actual exposure to AFs has been shown to be very high when considering maize and peanut products originating from Indonesia, with MOE values well below 10,000 [97], thus representing a major public health issue. Interestingly, since maize and peanut products are largely consumed in Indonesia, the MOE values remained below the accepted limits also when they were calculated for maize and peanut products produced in the EU [97]. Conversely, in Western countries, where stricter regulations are applied and the typical dietary pattern includes a smaller proportion of peanuts and tree nuts, the actual exposure to AFs is very low [98,99,100] and the potential benefits of nut consumption may outweigh the AF-related carcinogenic risk [100]. Indeed, Eneroth et al. showed that in a population of Swedish residents aged 55–79 years in 2013, with an average low exposure to aflatoxins, increasing the nut consumption from the current average of 5 g/day to 30 g/day could prevent 7680 individuals from developing a first CVD (306/100,000 person-years) and could contribute about 22,000 saved disability-adjusted life years (DALYs) for myocardial infarction [100]. At the same time, the potential increase in aflatoxin B1 exposure would lead to an estimated zero to three additional cases of liver cancer in the population, corresponding to approximately 159 DALYs. Thus, the population health benefits provided by increased nut consumption seemed to outweigh the risks associated with increased aflatoxin B1 exposure [99]. However, since these estimates were made upon data derived from the general population, it remains unclear whether a chronic exposure, even to low levels of AFs, may further increase the risk of HCC in subjects with an underlying fatty liver disease.

To the best of our knowledge, no studies have been conducted with the aim of evaluating a potential synergism between chronic AF-B1 exposure related to nut intake and the risk of HCC in the NAFLD population. Only one epidemiologic study conducted in the US evaluated the relation between nut consumption and HCC among 88,783 women from the Nurses’ Health Study and 51,492 men from the Health Professionals Follow-up Study [101]. After an average of 28 years of follow-up, nut consumption was not strongly associated with HCC risk (HR = 0.84, 95% CI: 0.56–1.26) though there was a suggestive inverse association with tree nut consumption. These results may refute the hypothesis that chronic nut intake might be harmful due to its intrinsic risk of AF-induced HCC tumorigenesis, at least in Western countries. Nevertheless, this study was not specifically aimed at evaluating AF contamination and therefore no data about AF exposure were provided. Furthermore, in the cohorts included, eventual concomitant liver diseases were not screened for, thus limiting the generalizability of these data for patients with an underlying liver disease. Thus, on these bases and pending potential studies that will provide an answer to the clinical question as to whether a potential AF contamination of nuts may lead to an enhanced risk of HCC in the already at-risk NAFLD population, it appears safe to recommend nut consumption in patients with NAFLD with the caveat that AF contamination should possibly be avoided completely, unless further data on safe thresholds for this special population are provided.

## 6. Conclusions

Nuts are health-promoting foods since they are rich in fibers, unsaturated fatty acids and antioxidants which help ameliorate lipid and glycemic metabolism and help reduce overall and cardio-vascular-related mortality. Therefore, these foods have been suggested as a useful nutritional tool for metabolic and weight control, and for reducing cardio-vascular mortality among subjects with NAFLD. However, strong evidence supporting this recommendation is still missing. Moreover, nuts can be contaminated by AF, a recognized carcinogenic agent, which can lead to the onset of HCC in patients with well-known risk factors. Safety regulations establishing the accepted limits for AF exposure have been set by the international Food Authorities based on risk-estimates calculated from data derived from the general population, and no data exist regarding the specific risk-estimates for subjects with underlying NAFLD. Whilst we cannot state that nut consumption may increase the risk for HCC in NAFLD unless solid evidence is available, we think that specialists taking care of these patients should inform them about the potential harms of nut intake when regularly including these foods in their dietary treatment. Since geographical origin may influence the risk of contamination, NAFLD-patients should be aware that foodstuffs coming from countries with stricter limits for AF contamination may be preferable in their case in order to prevent hepatotoxicity and hepatocellular carcinogenesis caused by AF-B1. On the other hand, we support the view that screening for AF contamination should be widely implemented using simple methods (e.g., UV absorption), mostly in nuts coming from areas at high risk of contamination, and long-term interventions at multiple levels should be sought. As an example, pre-harvest intervention may include the introduction of crops resistant to fungal infection or AF biosynthesis and the spraying of insecticides and fungicides. Besides, refraining from the use of plastic or synthetic materials (that promote humidity) for storage may be another useful preventing measure. Certainly, to maximize results, an integrated network of collaborations of different sectors, including public health, agricultural departments and mass media, is required to ensure effective food regulation systems and public awareness of the risks related to the chronic exposure to mycotoxins, especially among subjects with a pre-existing liver disease.

## Figures and Tables

**Table 1:** Beneficial effects of nuts.

Beneficial Effects of Nuts
- Reduced levels of LDL-cholesterol and triglycerides
- Reduced fasting and post-prandial glucose concentration
- Better weight control and weight reduction
- Improved satiety and reduced appetite
- Reduced risk of cardio-vascular disease and of cardio-vascular mortality
- Reduced risk of NAFLD

LDL, low-density lipo-protein; NAFLD, non-alcoholic fatty liver disease.

## Data Literature Search Strategy

Search strategy and selection criteria: we identified references for this review through a search of PubMed with the terms “steatosis”, “non-alcoholic fatty liver disease”, “nuts”, “hepatocellular carcinoma” and “aflatoxin” from 1 January 1991, to 30 September 2020. Only papers published in English were reviewed.
